# Supplementary material for: The Role of NK and T Cells in Endometriosis
Source: Int J Mol Sci. 2024 Sep 21;25(18):10141. doi: 10.3390/ijms251810141 (PMC11432446; doi:10.3390/ijms251810141)
Supplement: Supplementary file 1 [file ijms-25-10141-s001.zip › ijms-3183813-supplementary.pdf]

Table S1. List of antibodies used in the immunophenotyping panel.

Antibody	Fluorescence	Clone	Supplier
**CD3**	FITC	SK7	BD Biosciences, San Jose, CA, USA
**CD8**	PE-Cy7	SK1	Biolegend, Amsterdam, The Netherlands
**CD16**	APC-FireTM 750	3G8	Biolegend, Amsterdam, The Netherlands
**CD45**	PerCP	2d1	BD Biosciences, San Jose, CA, USA
**CD56**	BV421TM	HCD56	Biolegend, Amsterdam, The Netherlands
**CD57**	BV510 TM	QA17A04	Biolegend, Amsterdam, The Netherlands

APC, allophycocyanin; BV, brilliant violet; FITC, fluorescein isothiocyanate; PE, phycoerythrin;
PerCP, peridinin chlorophyll protein complex.

Table S2. Comparison of cells (T and NK) between Peripherical Blood and Peritoneal Liquid.

Parameter Statistics	Peripheral Blood (N = 62)	Peritoneal Liquid (N = 62)
**LY/WBC %**		
Mean (SD)	26.45 (10.464)	50.38 (18.278)
95% CI	[23.51; 29.39]	[45.23; 55.52]
Median	27.07	49.70
Median 95%CI	[24.38; 28.76]	[46.27; 57.48]
Q1, Q3	19.84, 33.12	37.83, 61.38
Min, Max	2.9, 51.5	1.6, 81.4
p-value [T]	<0.001	
**CD4 CD8- T cells (%)**		
Mean (SD)	66.44 (7.373)	35.53 (8.289)
95% CI	[64.37; 68.51]	[33.20; 37.86]
Median	67.36	35.63
Median 95%CI	[64.45; 70.33]	[33.21; 39.03]
Q1, Q3	61.72, 71.87	31.44, 41.40
Min, Max	47.0, 80.9	15.7, 54.6
p-value [T]	<0.001	
**CD4 CD16+ T cells (%)**		
Mean (SD)	2.41 (2.120)	21.19 (9.876)
95% CI	[1.89; 3.07]	[18.37; 24.14]
Median	1.96	18.94

Parameter Statistics	Peripheral Blood (N = 62)	Peritoneal Liquid (N = 62)
Median 95%CI	[1.58; 2.53]	[17.28; 23.16]
Q1, Q3	1.01, 3.19	14.72, 25.52
Min, Max	0.4, 13.4	4.1, 44.3
p-value [W]	<0.001	
**CD8 T cells (%)**		
Mean (SD)	33.56 (7.373)	64.47 (8.289)
95% CI	[31.49; 35.63]	[62.14; 66.80]
Median	32.64	64.38
Median 95%CI	[29.67; 35.55]	[60.96; 66.80]
Q1, Q3	28.14, 38.29	58.61, 68.56
Min, Max	19.1, 53.0	45.4, 84.3
p-value [T]	<0.001	
**CD8 CD56+ T cells (%)**		
Mean (SD)	23.62 (11.006)	53.69 (16.622)
95% CI	[20.57; 26.43]	[49.02; 58.37]
Median	21.35	53.72
Median 95%CI	[17.53; 26.30]	[47.34; 63.13]
Q1, Q3	13.69, 29.70	42.92, 67.33
Min, Max	7.9, 50.8	13.1, 85.6
p-value [T]	<0.001	
**CD56 T cells (%)**		
Mean (SD)	11.21 (5.569)	15.21 (2.994)
95% CI	[9.77; 12.80]	[14.36; 16.05]
Median	9.46	14.92
Median 95%CI	[8.37; 11.96]	[14.00; 16.29]

Parameter Statistics	Peripheral Blood (N = 62)	Peritoneal Liquid (N = 62)
Q1, Q3	7.30, 14.59	12.48, 17.01
Min, Max	3.6, 25.6	9.6, 21.5
p-value [T]	<0.001	
**CD56 CD16+ T cells (%)**		
Mean (SD)	26.76 (16.450)	39.13 (15.439)
95% CI	[22.14; 31.39]	[34.87; 43.40]
Median	25.67	35.28
Median 95%CI	[18.64; 30.73]	[31.91; 44.58]
Q1, Q3	10.90, 38.09	27.36, 53.19
Min, Max	1.1, 67.9	11.2, 66.8
p-value [T]	<0.001	
**NK cells (%)**		
Mean (SD)	14.66 (7.921)	14.08 (8.447)
95% CI	[12.64; 16.88]	[11.84; 16.36]
Median	11.81	12.81
Median 95%CI	[11.34; 16.08]	[8.29; 15.83]
Q1, Q3	9.47, 18.56	7.12, 19.03
Min, Max	3.7, 43.3	2.7, 33.3
p-value [T]	0.669	
**CD8+ TOTAL NK cells (%)**		
Mean (SD)	50.40 (12.372)	26.71 (9.206)
95% CI	[46.92; 53.88]	[24.32; 29.25]
Median	50.09	26.25
Median 95%CI	[46.24; 52.95]	[21.02; 30.01]
Q1, Q3	43.02, 58.87	19.15, 34.46

Parameter Statistics	Peripheral Blood (N = 62)	Peritoneal Liquid (N = 62)
Min, Max	16.6, 79.2	13.2, 48.0
p-value [T]	<0.001	
**CD4/CD8 Ratio**		
Mean (SD)	2.13 (0.702)	0.58 (0.205)
95% CI	[1.93; 2.32]	[0.52; 0.63]
Median	2.06	0.55
Median 95%CI	[1.81; 2.37]	[0.50; 0.64]
Q1, Q3	1.61, 2.55	0.46, 0.71
Min, Max	0.9, 4.2	0.2, 1.2
p-value [T]	<0.001	

N - number of subjects in the cohort;
SD - standard deviation; Q1 - 25th percentile; Q3 - 75th percentile;
[T] p-value calculated by means of an paired samples t-test; [W] p-value calculated by means of Wilcoxon signed rank test.

Table S3. Comparison of cells (T and NK) between Control and Endometriosis groups in Peripherical Blood and Peritoneal Liquid.

Parameter Statistcs	Peripheral Blood		Peritoneal Liquid	
	Control (N = 15)	Endometriosis (N = 47)	Control (N = 15)	Endometriosis (N = 47)
**T cells (cells/uL)**				
Mean (SD)	1050.51 (503.913)	1207.12 (370.373)	303.73 (324.698)	281.82 (207.993)
95% CI	[827.39; 1313.90]	[1098.38; 1315.87]	[165.40; 481.94]	[221.39; 350.13]
Median	1019.70	1188.55	187.39	229.48
Median 95%CI	[907.42; 1143.81]	[1071.92; 1288.27]	[77.62; 378.67]	[177.29; 306.95]
Q1, Q3	907.42, 1143.81	971.40, 1405.38	77.62, 378.67	146.31, 385.02
Min, Max	106.9, 2304.0	475.7, 2183.5	48.7, 1272.4	38.9, 1106.3
p-value	0.054		0.542	
**NK cells (%)**				
Mean (SD)	17.83 (9.731)	13.65 (6.663)	14.73 (10.095)	13.80 (7.805)
95% CI	[12.44; 23.22]	[11.81; 15.64]	[9.14; 20.32]	[11.44; 16.34]
Median	16.26	11.75	13.90	11.89
Median 95%CI	[11.81; 23.45]	[10.51; 15.32]	[5.76; 19.94]	[7.99; 16.29]
Q1, Q3	11.81, 23.45	9.29, 17.77	5.76, 19.94	7.29, 18.78
Min, Max	4.0, 43.3	3.7, 29.8	2.7, 33.3	4.6, 32.4
p-value	0.081		0.975	
**CD16Hi NK cells (%)**				
Mean (SD)	94.72 (2.905)	91.79 (7.858)	41.79 (15.299)	44.00 (16.255)

Parameter Statistcs	Peripheral Blood Control (N = 15)	Peripheral Blood Endometriosis (N = 47)	Peritoneal Liquid Control (N = 15)	Peritoneal Liquid Endometriosis (N = 47)
95% CI	[93.11; 95.91]	[89.54; 93.67]	[33.32; 50.26]	[38.88; 49.11]
Median	95.15	93.78	44.92	42.19
Median 95%CI	[93.98; 96.78]	[92.18; 94.79]	[28.89; 50.26]	[33.56; 53.09]
Q1, Q3	93.98, 96.78	91.17, 95.73	28.89, 50.26	29.37, 55.91
Min, Max	86.5, 97.3	57.5, 98.6	22.8, 73.2	22.5, 79.0
p-value	0.073		0.672	
**CD56Hi NK cells (%)**				
Mean (SD)	4.73 (2.716)	7.42 (5.889)	50.78 (17.610)	50.22 (19.533)
95% CI	[3.63; 6.36]	[5.96; 9.25]	[41.03; 60.53]	[43.61; 56.83]
Median	4.46	5.75	49.02	49.51
Median 95%CI	[3.21; 5.31]	[5.11; 7.72]	[41.78; 65.77]	[43.47; 63.50]
Q1, Q3	3.21, 5.31	4.51, 8.77	41.78, 65.77	39.25, 64.60
Min, Max	1.8, 13.4	1.7, 36.3	9.9, 77.0	17.4, 86.2
p-value	0.019		0.844	
**CD56Hi NK cells (cells/uL)**				
Mean (SD)	11.22 (13.143)	13.57 (10.402)	29.11 (29.603)	24.31 (27.465)
95% CI	[6.78; 19.14]	[11.03; 16.60]	[15.53; 43.22]	[16.22; 34.47]
Median	8.12	11.24	26.48	17.24
Median 95%CI	[5.58; 10.19]	[8.23; 12.64]	[2.23; 49.05]	[8.59; 22.44]
Q1, Q3	5.58, 10.19	7.24, 17.57	2.23, 49.05	5.49, 33.79
Min, Max	3.3, 57.4	3.5, 65.7	0.6, 104.9	1.5, 146.9

Parameter Statistcs	Peripheral Blood Control (N = 15)	Peripheral Blood Endometriosis (N = 47)	Peritoneal Liquid Control (N = 15)	Peritoneal Liquid Endometriosis (N = 47)
p-value	0.047		0.861	
**NK CD16Hi/NK CD56Hi Ratio**				
Mean (SD)	24.89 (11.642)	18.24 (11.718)	1.29 (1.772)	1.27 (1.179)
95% CI	[18.44; 31.33]	[15.07; 21.71]	[0.66; 2.22]	[0.91; 1.66]
Median	21.08	15.98	0.88	0.88
Median 95%CI	[17.79; 30.17]	[11.78; 18.52]	[0.41; 1.15]	[0.54; 1.34]
Q1, Q3	17.79, 30.17	10.47, 21.20	0.41, 1.15	0.48, 1.44
Min, Max	6.5, 53.4	1.7, 57.1	0.3, 7.4	0.3, 4.4
p-value	0.019		0.749	
**CD16Hi CD8+ NK cells (%)**				
Mean (SD)	50.25 (17.629)	52.66 (11.997)	34.10 (10.315)	42.50 (13.500)
95% CI	[40.48; 60.01]	[49.14; 56.19]	[28.39; 39.81]	[38.49; 46.51]
Median	50.47	52.96	35.48	42.34
Q1, Q3	40.25, 63.80	44.96, 60.95	23.14, 40.01	32.97, 51.87
Min, Max	16.0, 80.3	26.0, 79.5	18.9, 52.0	17.7, 68.1
p-value	0.681		0.029	

N - number of subjects in the cohort; SD - standard deviation; Q1 - 25th percentile; Q3 - 75th percentile
